# Postoperative Leckagen im Gastrointestinaltrakt – Diagnostik und Therapie

**DOI:** 10.1007/s11377-021-00584-6

**Published:** 2022-01-10

**Authors:** G. Loske, S. Hornoff, M. Mende, C. Müller, S. Faiss

**Affiliations:** 1 Klinik für Allgemein‑, Viszeral‑, Thorax- und Gefäßchirurgie, Kath. Marienkrankenhaus gGmbH Hamburg, Alfredstraße 9, 22087 Hamburg, Deutschland; 2 Klinik für Innere Med. I mit Schwerpunkt Gastroenterologie, Sana-Klinikum Berlin-Lichtenberg, Fanningerstraße 32, 10365 Berlin, Deutschland

**Keywords:** Anastomoseninsuffizienz, Endoskopie, Stents, Unterdrucktherapie, Drainage, Anastomotic insufficiency, Endoscopy, Stents, Negative pressure therapy, Drainage

## Abstract

Postoperative Leckagen nach Ösophagus‑, Magen- oder Kolon- bzw. Rektumchirurgie stellen schwerwiegende chirurgische Komplikationen mit einer hohen Morbidität und Mortalität dar. Leckagen werden zumeist durch eine Kombination aus klinischer Beobachtung, Infektionsparametern sowie endoskopischen und schnittbildgebenden Verfahren diagnostiziert. Die Therapie ist bei intraperitonealen Leckagen chirurgisch, bei retroperitonealen bzw. mediastinalen Leckagen in einem interdisziplinären Setting überwiegend interventionell endoskopisch. Hier stehen der Defektverschluss durch die Abdichtung mittels selbstexpandierender gecoverter Stents mit gleichzeitiger externer extraluminaler Drainage sowie der Defektverschluss mit simultaner innerer Drainage und endoskopischer Unterdrucktherapie als vorrangige Methoden zur Verfügung.

## Abstract (en)

Postoperative leaks following esophageal, gastric, colonic or rectal resection represent a life-threatening complication that is associated with high morbidity and mortality. Leaks are generally diagnosed with a combination of the following: recognition of clinical deterioration, laboratory results, endoscopic and radiological imaging. In case of intraperitoneal leaks, surgery is mandatory. In case of mediastinal or retroperitoneal anastomotic insufficiencies, treatment mainly comprises interventional endoscopic procedures in an interdisciplinary approach. For endoscopic therapy, there are mainly two different methods described: closure of the defect with fully covered self-expandable stents with external drainage and closure of the defect with simultaneously internal drainage by endoscopic vacuum therapy.

## Lernziele

Nach Lektüre dieses Beitrags …

- können Sie die Diagnostik postoperativer Leckagen im Gastrointestinal(GI)-Trakt zuverlässig anwenden;
- können Sie die unterschiedlichen diagnostischen Verfahren in ihrer Wertigkeit entsprechend einordnen;
- kennen Sie die unterschiedlichen Verfahren zur Therapie postoperativer Leckagen im GI-Trakt;
- kennen Sie die Vor- und Nachteile dieser unterschiedlichen Verfahren;
- können Sie diese Verfahren entsprechend den unterschiedlichen Leckagetypen einordnen und differenziert einsetzen.

## Hintergrund

Anastomoseninsuffizienzen (AI) des GI-Trakts stellen schwerwiegendste chirurgische Komplikationen dar, die zu einer deutlich erhöhten Morbidität und **Mortalität**

Mortalität

des Eingriffs führen. Verschiedene interventionell-endoskopische Verfahren zur Therapie der AI stehen zur Verfügung. Es wird im Folgenden im Wesentlichen auf postoperative Leckagen (PL) nach Ösophagus‑, Magen- und Rektumchirurgie eingegangen.

## Diagnostik

Je nach regionaler Lokalisation der betroffenen Naht sind die Auswirkungen einer PL unterschiedlich. Bei intrathorakalen Anastomosen drohen die **Mediastinitis**

Mediastinitis

und das Pleuraempyem. Bei Beteiligung der Abdominalhöhle ist die **Peritonitis**

Peritonitis

und im extraperitonealen kleinen Becken die septische Phlegmone eine gefürchtete Folge. Dementsprechend unterscheiden sich die klinischen Auswirkungen. Zur Diagnostik stehen generell klinische und laborchemische Parameter sowie bildgebende Verfahren (Endoskopie, Röntgenkontrastverfahren, Schnittbildgebung) zur Verfügung. Die frühzeitige Identifizierung senkt die Morbidität und Mortalität [1, 2].

### Klinische Parameter

Die PL kann sich klinisch hinter einer Vielzahl von Symptomen verbergen. Jede postoperative klinische Verschlechterung (Tachykardie, Tachypnoe, Hypotension, Fieber, Paralyse, Verwirrtheit bis hin zur **Sepsis**

Sepsis)

muss an eine PL denken lassen und erfordert eine entsprechende unverzügliche Diagnostik zum Ausschluss. Der Wechsel von Drainageflüssigkeiten hin zu **pathologischen Sekreten**

pathologischen Sekreten

(gallig, stuhlig, putride) sind eindeutige Zeichen. Kontaminierte extraluminale **Flüssigkeitsverhalte**

Flüssigkeitsverhalte

führen zu erhöhten laborchemischen Infektionszeichen.

#### Merke

Die klinische Verschlechterung eines postoperativen Patienten kann Hinweis auf eine postoperative Leckage sein.

### Laborchemische Parameter

Hier sollte vor allem der postoperative Verlauf der **Infektionsparameter**

Infektionsparameter

(C-reaktives Protein [CRP], Leukozyten, Prokalzitonin) beachtet werden. Insbesondere ein Anstieg des CRP ist verdächtig und ein frühes Zeichen der PL.

### Endoskopie

Die flexible endoskopische Untersuchung ist ein fester und unverzichtbarer Bestandteil im Management der PL. Die direkte endoskopische Untersuchung ist das wesentliche Verfahren, um die Anastomose zu untersuchen [3], und kann heutzutage als der **Goldstandard**

Goldstandard

der Diagnostik angesehen werden. Frühere Bedenken, man könne durch die diagnostische Endoskopie eine iatrogene Schädigung an der Anastomose herbeiführen, sind in den letzten Jahren einem klaren Paradigmenwechsel hin zur Endoskopie gewichen. In den meisten Fällen kann eine PL direkt detektiert werden. Die Endoskopie lässt sich sowohl in den Endoskopieräumen also auch im Operationssaal, auf der Intensivstation und mobil „bedside“ durchführen. Sie kann mit einer **Röntenkontrastuntersuchung**

Röntenkontrastuntersuchung

kombiniert werden. Zum Einsatz kommen bevorzugt diagnostische und kleinkalibrige Endoskope. Aufgrund der rascheren Resorption, geringeren Ballonierung und des potenziellen Risikos der Luftembolie ist CO_2_ als Untersuchungsgas zu bevorzugen [4].

Beim Nachweis einer PL spielt die Endoskopie für die weitere Therapieplanung die entscheidende Rolle (siehe im Folgenden). Die Defektgröße, eine extraluminale **Infekthöhle**

Infekthöhle,

die Durchblutungssituation bzw. eine Ischämie und die daraus folgende Nekrose lassen sich bildlich exakt darstellen. Anhand der erhobenen Befunde ist zu entscheiden, welches Therapieverfahren angewendet werden soll. Eine endoskopische Therapiemaßnahme kann sich in derselben Untersuchung unmittelbar ohne weiteren Zeitverlust anschließen.

Nur endoskopisch kann beurteilt werden, ob eine lokale **Wundheilungsstörung**

Wundheilungsstörung

der Anastomose vorliegt, die im weiteren Verlauf in einer PL enden kann. Sichtbares freiliegendes Naht- und Klammermaterial, breite Ulzerationen und Durchblutungsstörungen sind wichtige Indikatoren einer **Risikoanastomose**

Risikoanastomose

(At-risk-Anastomosen, ARA; [5, 6]). Bei Anastomosen mit diesen Risikokriterien empfehlen sich engmaschige Kontrolluntersuchungen bzw. die unmittelbare Einleitung einer präemptiven intraluminalen **endoskopischen Unterdrucktherapie**

endoskopischen Unterdrucktherapie

(EUT; [7, 8]).

#### Merke

Die Endoskopie spielt eine wesentliche Rolle in der Diagnostik früher Anastomoseninsuffizienzen.

### Röntgenkontrastverfahren

Die indirekte Darstellung einer PL mittels Röntgenkontrastmittel (z. B. postoperative Ösophagusdarstellung mit wasserlöslichem Kontrastmittel) ist ein gutes und sicheres Verfahren zur Darstellung auch kleinster PL durch **extraluminale Kontrastmittelaustritte**

extraluminale Kontrastmittelaustritte.

Sie kann mit der Endoskopie kombiniert werden. Im Unterschied zur Endoskopie wird die PL aber erst bei einem manifesten **transmuralen Defekt**

transmuralen Defekt

diagnostiziert. Aussagen zur Durchblutungssituation oder zum Vorliegen einer kritischen Anastomosensituation (ARA) können nicht getroffen werden [9]. Das bedeutet, dass die Röntgendarstellung eine noch intakte Anastomose zeigen kann, während endoskopisch bereits eine ARA vorliegt. Im Vergleich zu Schnittbildverfahren zeigt die indirekte Röntgenkontrastdarstellung nicht die Umgebung und das ganze Ausmaß der Leckage [10].

### Schnittbildgebende Computertomographie

Die schnittbildgebende Computertomographie (CT) ermöglicht die unverzügliche Untersuchung der Anastomosenregion mit gleichzeitiger Abbildung der extraluminalen Umgebung. Sie detektiert nicht nur die PL selber, sondern auch das periluminale Ausmaß der Leckage (z. B. Größe eines Abszesses) und die **umgebenden Begleiterscheinungen**

umgebenden Begleiterscheinungen

(z. B. Mediastinitis, freie Luft, Flüssigkeitsverhalte; [1]). Es wird die Durchführung mit intravenösem und intraluminalem Kontrastmittel empfohlen.

Die CT ist der konventionellen Röntgenkontrastdarstellung überlegen, aber auch hier ist zu beachten, dass erst beim Vorliegen eines manifesten transmuralen Defekts dieser eindeutig gesichert werden kann. **Extraluminale Gaseinschlüsse**

Extraluminale Gaseinschlüsse

sind hochverdächtig auf das Vorliegen einer PL und müssen zügig durch eine Endoskopie überprüft werden.

Die CT hat noch eine besondere Bedeutung im Laufe einer eingeleiteten endoskopischen Therapiemaßnahme. Nicht ausreichend drainierte Flüssigkeitsverhalte können detektiert werden und einer **interventionellen Drainage**

interventionellen Drainage

zugeführt werden.

### Resümee

Leckagen werden zumeist durch eine Kombination aus klinischer Beobachtung, Infektionsparametern, endoskopischer und schnittbildgebender Verfahren diagnostiziert. Bei einem klinischen Verdacht sollten eine CT zur Bestätigung und eine endoskopische Darstellung der Anastomose erfolgen. Bei frühen Formen einer Insuffizienz kann eine CT noch einen normalen Befund ergeben, eine Endoskopie jedoch schon den Anastomosendefekt oder eine Wundheilungsstörung im Sinne einer ARA direkt visualisieren [10, 11].

## Therapie

Sämtliche Therapiekonzepte beim Vorliegen einer PL haben die Beseitigung bzw. Verhinderung der Komplikationsfolgen zum Ziel. Zu beachten ist, dass 2 wesentliche chirurgische Grundprinzipien eine unverändert hohe Bedeutung haben: der **Defektverschluss**

Defektverschluss

und die **Sekretableitung**

Sekretableitung.

Die Behandlungen sind schwierig und erfordern häufig eine enge interdisziplinäre Zusammenarbeit mit Ausschöpfung aller intensivmedizinischen Ressourcen.

Die Endoskopie liefert die wichtigsten Informationen zur Therapieplanung der PL. Im Idealfall kann sofort nach der diagnostischen Untersuchung die endoskopische Therapie eingeleitet werden. Anhand der erhobenen Befunde ist zu entscheiden, ob und welche endoskopische Behandlung erfolgen kann oder ob eine operative Revision erfolgen muss.

### Überlegungen zur chirurgischen Therapie

Chirurgisch operative Maßnahmen zur Therapie einer Leckage am GI-Trakt erfordern in der Regel, dass die bereits verschlossene oberflächliche und intrakorporale Wunde wiederöffnet wird. Gelingt es nicht, eine ausreichend effiziente Drainage zu installieren und den Defekt zu verschließen, kann sich die Kontamination in vorher unbeteiligte Körperregionen ausbreiten. Die radikalste chirurgische Maßnahme nach kolorektalen Resektionen ist die **Diskontinuitätsoperation**

Diskontinuitätsoperation,

d. h. die Resektion des Anastomosenbereichs mit Ausleitung des Darminhalts über ein Stoma, um die fortwährende Kontamination zu unterbrechen. Je nach Lokalisation erfolgt dieses nach Ösophagusresektion in Form einer ösophagealen Speichelfistel mit distalem Blindverschluss, nach Darmresektionen mittels Anlage einer Jejuno‑, Ileo- oder Kolostomie. Nach Ausheilung sind aufwendige **Revisionsoperationen**

Revisionsoperationen

notwendig, um die Kontinuität wiederherzustellen.

Bei sehr frühen technisch bedingten AI und Insuffizienzen, die auf einer fulminanten Ischämie des betroffenen Intestinalabschnitts beruhen (Magenconduitnekrose, Kolonischämie), ist die chirurgische Revision zwingend erforderlich. Entleert sich Darminhalt in die freie Bauchhöhle, ist eine Laparotomie unumgänglich.

### Pathophysiologische Besonderheiten der postoperativen Leckage

Es ist zu beachten, dass bei einer PL sowohl der eigentliche lokale Darmwanddefekt als auch die extraluminale Infektion behandelt werden müssen. Oft ist zu beobachten, dass der endoskopisch zu detektierende Wanddefekt klein ist, dahinter aber eine größere extraluminale **Wundhöhle**

Wundhöhle

liegt, die den eigentlichen septischen Fokus darstellt. Der endoskopisch sichtbare Defekt ist häufig „nur die Spitze des Eisbergs“. Mit kleinlumigen Endoskopen kann der Defekt überwunden und die extraluminale Wundhöhle inspiziert werden.

### Vorteil der endoskopischen Therapie und geeignete Therapieorte

Der große Vorteil der Endoskopie ist, dass das intestinale Operationsgebiet über die natürlichen Körperöffnungen von Mund und Anus von luminal erreicht werden können. Zur Beurteilung der Wundverhältnisse muss das Wundgebiet von außen nicht wiedereröffnet werden. Bei allen PL, die endoskopisch zu sehen sind, kann daher eine endoskopische **lokale Therapiemaßnahme**

lokale Therapiemaßnahme

erwogen werden. Eine weitere Grundvoraussetzung ist, dass sich der Defekt nicht in die freie Abdominalhöhle oder Pleurahöhle entleert. Geeignete Lokalisationen für die endoskopische Behandlung sind daher alle Räume, die schon physiologischerweise von der freien Körperhöhle getrennt sind. Hierzu gehören: das extraperitoneale Becken, Mediastinum und Retroperitoneum.

#### Merke

Bei allen postoperativen Leckagen, die endoskopisch zu sehen sind, kann eine endoskopische lokale Therapiemaßnahme erwogen werden.

### Endoskopische Techniken

Die beiden wichtigsten endoskopischen Techniken, die zur Therapie von PL zum Einsatz kommen, sind:

1. Defektverschluss durch Abdichtung mittels selbstexpandierende gecoverte Stents (SCS) und gleichzeitiger externer extraluminaler Drainage;
2. Defektverschluss und gleichzeitige innere Drainage mittels EUT.

#### Merke

Zur endoskopischen Therapie stehen vor allem 2 Techniken zur Verfügung: Stentverschluss und endoskopische Unterdrucktherapie.

Beide Methoden unterscheiden sich in den zugrunde liegenden technischen Wirkprinzipien, die dargestellt werden sollen.

### Defektverschluss durch Abdichtung mittels gecoverten Stents

Der Einsatz von SCS ist eines der häufigsten angewandten endoskopischen Verfahren zur Therapie einer postoperativen Leckage im oberen GI-Trakt [12]. Diese Möglichkeit der Defektabdichtung mit obligater Lokaldrainage des extraluminalen Fokus durch eine externe Drainage ist ein schon lange etabliertes Konzept. Die notwendigen operativ oder interventionell eingebrachten Drainagen dienen der extraluminalen Sekretableitung, um die Entstehung einer **extraluminalen Abszedierung**

extraluminalen Abszedierung

oder Phlegmone zu verhindern.

**Figure 1:**
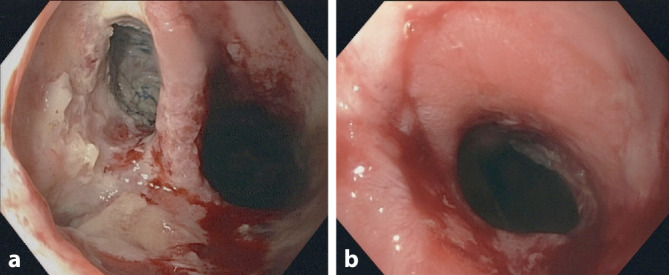

Die SCS kommen bei postoperativen Drainagen im oberen GI-Trakt sowohl nach resezierenden **onkologischen Eingriffen**

onkologischen Eingriffen

(Ösophagusresektionen, Gastrektomie) als auch bariatrischen Operationen zum Einsatz. Die klinische Erfolgsrate wird von 70 bis zu 90 % angegeben (Abb. 1; [13]).

Das Behandlungsprinzip besteht darin, dass sich der SCS nach der Freisetzung im Intestinallumen entfaltet und expandiert. Mit der flüssigkeitsdichten Wandung schmiegt er sich von innen an die Intestinalwand. Bei großlumigen Intestinalabschnitten oder Anastomosenregionen mit einer Lumeninkongruenz, wie z. B. nach **abdominothorakaler Ösophagusresektion**

abdominothorakaler Ösophagusresektion,

ist dieses häufig nur unzureichend möglich. Limitationen sind auch bedingt durch die Lokalisation des Behandlungsorts (zervikaler Ösophagus/Kardia). Das Ziel der Beendigung der Kontamination durch Verdauungssekrete kann dann nicht oder nur inkomplett erreicht werden.

Je nach ursprünglicher Größe der Insuffizienz wird der SCS nach 4–8 Wochen wieder entfernt. Die Behandlung ist mit zum Teil erheblichen Komplikationen belastet (Migration, Perforation, Arrosion, Blutung, fehlende Entfernbarkeit, mangelnde Abdichtung). Ein häufiges Problem stellt die hohe **SCS-Dislokationsrate**

**Figure 2:**
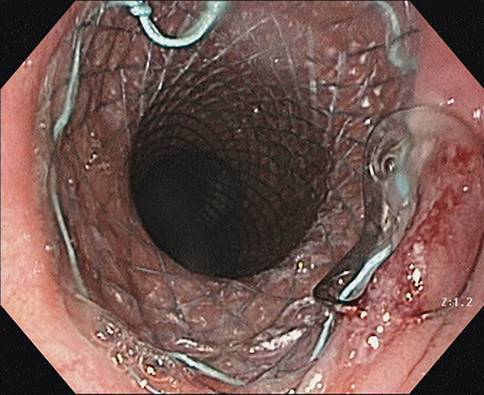

SCS-Dislokationsrate

von 8–20 % dar, die neuerdings durch die Fixierung mit speziellen Clips unterbunden werden soll (Abb. 2; [14]).

### Defektverschluss und gleichzeitige innere Drainage mit endoskopischer Unterdrucktherapie

Bei der EUT wird mittels offenporiger Drainagen und elektronischer Vakuumpumpen ein kontinuierlicher Unterdruck an den inneren Wundbereich angelegt [15]. Die Behandlung erfolgt über mehrere Tage, die Drainagen müssen im Abstand von einigen Tagen regelmäßig gewechselt werden. Empfohlen werden **Wechselintervalle**

Wechselintervalle

von 3–4 Tagen. Dabei wird auch eine endoskopische Inspektion der inneren Wunde vorgenommen und die Behandlung an die Wundverhältnisse adaptiert.

#### Intrakavitäre und intraluminale Varianten der EUT

Bei der intrakavitären EUT wird das offenporige Drainageelement durch den Intestinaldefekt hindurch in der extraluminalen Wundhöhle platziert. Mit Ausübung eines permanenten Unterdrucks wird die Höhle leergesaugt, sodass sie kollabiert. Bei der intraluminalen Therapievariante wird die offenporige Drainage im Intestinallumen in Höhe des Defekts und diesen überdeckend platziert. Bei **Sogausübung**

Sogausübung

kollabiert das Lumen. Auf diese Weise soll eine Abdichtung des Defekts erzielt werden. Intrakavitäre und intraluminale Varianten [16] lassen sich miteinander kombinieren. Das Ziel ist es, eine sofortige Abdichtung des Defekts mit gleichzeitiger nach intraluminal gerichteter Drainagewirkung herbeizuführen.

#### Unterdruckdrainagen mit offenporigem Polyurethanschaum und doppellagiger offenporiger Folie

Bislang wurden als offenporiges Drainagematerial Polyurethanschäume (PUS) mit einer Porengröße zwischen 400 und 600 µm verwendet. Die PUS zeichnen sich durch eine gute Drainagewirkung, Soganhaftung auf Gewebe und **Débridement**

Débridement

der Wundoberfläche aus. Ein technischer Nachteil kann das Volumen des PUS-Materials darstellen, das einen konstruktiven Drainagendurchmesser von mehr als 1,5 cm bedingt. Bei kleinen Öffnungen kann daher die intrakavitäre Platzierung unmöglich sein.

**Figure 3:**
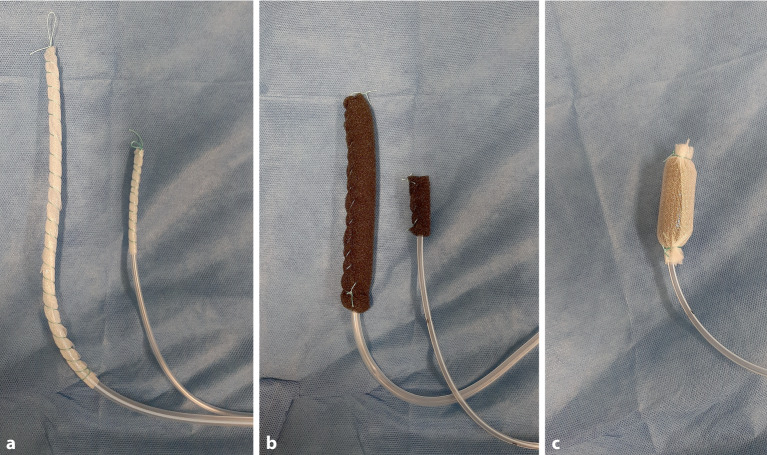

Neue **folienbasierte Drainagen**

folienbasierte Drainagen

wurden entwickelt, bei denen das Drainageelement aus einer dünnen offenporigen doppellagigen Membran besteht [17]. Diese Drainagen sind gut geeignet, flüssige Sekrete abzuleiten, sie haften nicht so stark auf dem Wundgrund. Aufgrund des kleinen Durchmessers lassen sie sich in kleine Defektöffnungen und transnasal, wie eine Magenableitsonde, einführen. Die Abb. 3 gibt einen Überblick über Grundtypen von schwamm‑ und folienbasierten Unterdruckdrainagen, die bei der EUT zur Anwendung kommen.

**Figure 4:**
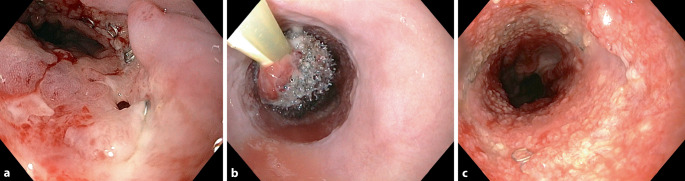

Die meisten Erfahrungen mit der EUT liegen für Ösophagus (Abb. 4; [18, 19]) und Rektum [20] vor. Erfolgreiche Anwendungen erfolgten auch im Duodenum [21] und nach bariatrischer Chirurgie [22, 23].

Ein wesentlicher Unterschied zur endoskopischen Stenttherapie besteht darin, dass es sich um eine innere **chirurgische Wundtherapie**

chirurgische Wundtherapie

handelt, die mit einem regelmäßigen endoskopischen Drainagenwechsel, meist im Abstand von 3–4 Tagen, verbunden ist. Die innere Wunde muss begutachtet und die Therapie entsprechend angepasst werden. Alle offenporigen Drainagen können verstopfen und dann funktionslos werden. Bei der Behandlung im Rektum sind aufgrund der möglichen Verstopfung mit Kot oft die Anlage eines **Anus praeter**

Anus praeter

und die Spülung des Kolons notwendig.

Eine relevante seltene Komplikation bei der intrakavitären thorakalen Anwendung der EUT mit PUS stellen Blutungen dar. Einige Autoren schlagen daher die Überprüfung mittels CT vor. Die CT hat auch als Verlaufsuntersuchung eine besondere Bedeutung, um extraluminale, nichtdrainierte Flüssigkeitsverhalte auszuschließen.

### Vergleich von SCS und EUT bei Ösophagusdefekten

Mittlerweile liegen erste Metaanalysen [24] vor, die SCS und EUT miteinander vergleichen. Diese fassen die Ergebnisse von mehreren retrospektiven Studien zusammen [25]. Bei den meisten Patienten besteht eine AI nach Ösophagektomie. Für die EUT wurden Vorteile in der Höhe der Heilungsrate, eine geringere Rate an Major-Komplikationen, eine geringere Mortalitätsrate und eine kürzere Behandlungsdauer nachgewiesen.

Auch für den Einsatz der EUT im Vergleich zur Stentbehandlung bei Defekten nach bariatrischer Chirurgie liegen erste Daten vor, die einen Vorteil für die EUT zeigen [23].

Randomisierte prospektive Studien fehlen. Die SCS sind seit Jahren ausgereifte **zugelassene Medizinprodukte**

zugelassene Medizinprodukte,

während für die EUT derzeit nur 2 zugelassene Drainagen erhältlich sind. Neue Typen von Unterdruckdrainagen zeigen, dass die technische Entwicklung für die EUT noch nicht abgeschlossen ist. Um ein optimales Behandlungsergebnis zu erzielen, muss der Anwender bei der EUT die geeigneten Materialien kreativ adaptieren. Speziell für die EUT entwickelte und zugelassene Unterdruckpumpen sind nicht verfügbar. Hieraus resultiert, dass in vielen Fällen der EUT-Anwendung formal keine **Medizinproduktsicherheit**

Medizinproduktsicherheit

gegeben ist. Umso erstaunlicher ist es, dass die EUT im Komplikationsmanagement in wenigen Jahren diesen hohen Stellenwert erreicht hat. Gemeinsam mit den Partnern der Industrie muss nach Lösungen gesucht werden, damit zugelassene Medizinprodukte den Anwendern zur Verfügung stehen [26].

### „Stent-over-sponge“ und Vakuumstent

Zwei unterschiedliche Methoden versuchen beide Verfahren vorteilhaft miteinander zu verbinden. Bei der Stent-over-sponge(SOS)-Methode wird der Defekt zusätzlich zu einer **intrakavitären Unterdruckdrainage**

intrakavitären Unterdruckdrainage

mit einem gecoverten Stent überdeckt. Hierdurch soll eine bessere Abdichtung des Kompartiments, das unter den Unterdruck gesetzt wird, erreicht werden [27]. Des Weiteren liegt bereits als ein zugelassenes Medizinprodukt ein selbstexpandierender kurzer Stent vor, dessen Außenwand mit einem offenporigen Schaum ummantelt ist und mit einem Drainageschlauch unter einen Unterdruck gesetzt werden kann. Durch diese Anordnung soll eine intraluminale Unterdrucktherapie mit Abdichtung des Defekts bei gleichzeitig erhaltener Passage gewährleistet werden. Erste Erfahrungen zur Anwendung liegen vor [28].

### Präemptive EUT bei Ösophagusanastomosen

Auch wenn der Fokus des Beitrags auf die Diagnostik und Therapie von bestehenden PL steht, soll darüber hinaus auf eine zusätzliche Indikation der EUT hingewiesen werden. Erste Studien zeigen, dass eine präemptive intraluminale EUT bei der abdominothorakalen Ösophagusresektion einen wesentlichen Beitrag zur Verhinderung einer AI leisten kann.

Im Jahr 2017 haben Neumann et al. in einer kleinen Fallserie die präemptive Indikationsstellung bei Ösophagusresektion vorgestellt, nachdem schon zuvor in Einzelfällen hiervon berichtet wurde [7]. In frühen postoperativen Kontrollendoskopien bei 8 Patienten ihrer Fallserie fanden sie umschriebene Ischämien der Anastomosenregion. Die für eine PL als gefährdet eingeschätzte Nahtregion wurde vorbeugend mit intraluminalen **Polyurethanschaumdrainagen**

Polyurethanschaumdrainagen

überdeckt und die intraluminale EUT eingeleitet. Bei allen Patienten kam es zur Abheilung der Anastomose.

Eine noch weitergehende Indikation zur präemptiven Therapie bei abdominothorakaler Ösophagusresektion wurde im Jahr 2021 von 2 Arbeitsgruppen vorgelegt, die mit der präemptiven Therapie bereits intraoperativ nach Fertigung der Anastomose beginnen. Müller et al. berichten über die Erfahrung mit mittlerweile 67 Patienten [5], bei denen eine intraluminale EUT mit einer Polyurethanschaumdrainage in Höhe der Anastomose unmittelbar nach Fertigung der Nahtverbindung für eine Dauer von 4–6 Tagen durchgeführt wurde. Bei 73 % der Patienten war die Anastomosenheilung unkompliziert, bei 27 % wurde die EUT bei Heilungsstörungen an der Anastomose fortgeführt.

Loske et al. berichten über eine erste Patientenserie mit 24 Patienten mit **Ivor-Lewis Resektionen**

Ivor-Lewis Resektionen,

bei denen präemptiv, intraoperativ beginnend, eine intraluminale Unterdrucktherapie eingeleitet wurde. Sie nutzen eine dünne doppellumige offenporige Foliendrainage (OFD), die transnasal eingeführt und im Magen platziert wird. Nach Anlegen eines permanenten Unterdrucks wird der Magen dauerhaft leergesaugt. Ziel ist es, in der ersten vulnerablen Phase der Wundheilung die Anastomosenregion frei von den Verdauungssäften des Refluxes zu halten [6]. Die Drainage hat einen Durchmesser von 6 mm, sodass sie wie eine Magensonde transnasal eingeführt werden kann. Gleichzeitig zur Absaugung ist über eine integrierte **Intestinalsonde**

Intestinalsonde

die enterale Ernährung möglich. Bei 24 Patienten wurde eine Anastomosenheilungsrate von 100 % erzielt. Wundheilungsstörungen an der Anastomose wurde bei 10 von 24 Patienten gesehen, die unter einer prolongierten Refluxdrainage zur vollständigen Abheilung kamen. Die Autoren vermuten, dass der postoperative Reflux einen wesentlichen Faktor für die Entstehung von AI darstellte, der mit der Methode beseitigt werden kann.

Beide Studien lassen darauf schließen, dass sich die Rate an symptomatischen und interventionsbedürftigen AI durch die intraluminale EUT mit Schwamm oder Foliendrainagen reduzieren lässt. Eine weitere Evaluation der präemptiven und prophylaktischen EUT ist von großem Interesse.

### Andere endoskopische Verfahren

Andere endoskopische Verschlussfahren mit Clips oder Naht spielen bei der primären Versorgung der postoperativen Leckage eine untergeordnete Rolle. Dieses ist insbesondere durch die Infektion an der **Anastomosennaht**

Anastomosennaht

bedingt, die in der Regel nicht nahtfähig ist. Allenfalls bei wenige Stunden alten Insuffizienzen bei fehlender Infektion und guter Durchblutung könnte eine Defektbehebung mit einem Clip erfolgreich sein. Bei einem frühen technischen Nahtversagen sollte aber auch die sofortige chirurgische Revision bedacht werden.

### Resümee

Die endoskopischen Behandlungsmethoden haben bei der postoperativen Leckage des GI-Trakts einen sehr hohen Stellenwert erlangt. Die beiden wesentlichen endoskopischen Therapieoptionen stellen die Verwendung von SCS und die EUT dar. Die wesentliche Neuerung zur Behandlung der postoperativen Leckage ist die EUT. Diese Methode vereint die beiden wichtigen chirurgischen Behandlungsprinzipien von Defektverschluss und Drainage.

Für die abdominothorakale Ösophagusresektion liegen erste Studien vor, die zeigen, dass die intraluminale EUT mit Schwamm- oder Foliendrainagen präemptiv genutzt werden kann. Hierdurch soll die Rate an postoperativen Leckagen und Anastomoseninsuffizienzen reduziert werden.

## Fazit für die Praxis

- Die Diagnose von postoperativen Leckagen ergibt sich aus der Kombination von klinischer Beobachtung, Endoskopie, Computertomographie und Infektionsparametern.
- Bei einem klinischen Verdacht auf eine Anastomoseninsuffizienz sollte ohne Zeitverzug eine endoskopische Darstellung der Anastomose erfolgen.
- Mit der Endoskopie kann eine Wundheilungsstörung an der Anastomose im Sinne einer Risikoanastomose (ARA) mit drohender Anastomoseninsuffizienz frühzeitig direkt visualisiert werden.
- Die beiden wichtigsten endoskopischen Methoden zur Therapie einer postoperativen Leckagen sind die Verwendung von selbstexpandierenden Metallstents (SCS) und die endoskopische Unterdrucktherapie (EUT).
- Die EUT mit offenporigen Schwamm- oder Foliendrainagen kann bei der Ösophagektomie präemptiv zur Anastomoseninsuffizienzprophylaxe eingesetzt werden.

## CME-Fragebogen

Welche diagnostischen Mittel führen zu einer sicheren Diagnose einer postoperativen Leckage?

Die Endoskopie ist das diagnostische Verfahren der Wahl.

Klinische Symptome des Patienten beweisen eine postoperative Leckage.

Leckagen werden zumeist durch eine Kombination aus klinischer Beobachtung, Infektionsparametern, endoskopischer und schnittbildgebender Befunde diagnostiziert.

Die Computertomographie ist das diagnostische Mittel der Wahl.

Sonographie und Endoskopie ermöglichen eine sichere Diagnosestellung einer postoperativen Leckage.

Welche schwerwiegende Komplikation kann eine postoperative Leckage nach einer abdominothorakalen Ösophagusresektion mit intrathorakaler Anastomosierung haben?

Peritonitis

Appendizitis

Septische Phlegmone

Erysipel

Mediastinitis

Welche Funktion und welche diagnostische Aussage kann die Endoskopie bei einer postoperativen Leckage erbringen?

Die Endoskopie kann den luminalen Wanddefekt hinsichtlich seiner Größe und Lokalisation exakt bestimmen.

Die Endoskopie kann die Umgebung und das ganze Ausmaß der Leckage darstellen.

Die Endoskopie kann im Gastrointestinaltrakt diagnostisch und therapeutisch nur bei Ösophagusleckagen eingesetzt werden.

Die Endoskopie muss durch ein Computertomographie ergänzt werden, um die postoperative Leckage zu finden.

Die Endoskopie bietet *kein*e therapeutische Option bei postoperativen Leckagen.

Welche *un*erwünschten Effekte können nach Stentimplantation auftreten?

Dislokation, Blutung und Obstruktion

Blutung und Perforation

Perforation und Infektion

Dislokation und Perforation

Dislokation, Blutung, Perforation, inkomplette Abdichtung

Was ist das Prinzip der endoskopischen Unterdrucktherapie?

Abdichtung des Defekts mit gleichzeitige Sekretdrainage durch Unterdruck

Sogumkehr des Druckgradienten von endoluminal nach intramediastinal/-thorakal durch Unterdruckanlage

Verschluss der Leckage durch Vakuumspülung des Lumens

Abdichtung der Anastomoseninsuffizienz durch Verstopfen mit einem Schwamm

Hemmung der Motilität und damit Verschluss der Anastomoseninsuffizienz

Welchen Nebeneffekt erzielt die intraluminale endoskopische Unterdrucktherapie bei einer Anastomoseninsuffizienz nach abdominothorakaler Ösophagusresektion?

Reduktion der Entzündung durch lokale Antibiotikaabgabe

Verbesserung der oralen Nahrungsaufnahme

Drainage des Refluxes und damit „Trockenlegen“ der Anastomosenregion

Ständige Befeuchtung der Anastomosenregion

Es gibt keine Nebeneffekte

Welches diagnostische Mittel ist am besten geeignet, um bei einer intrakavitären EUT Komplikationen und *nicht* ausreichend drainierte Flüssigkeitsverhalte *aus*zuschließen?

Sonographie

Endoskopie

Computertomographie

Positronenemissionstomographie und Computertomographie (PET-CT)

Ösophagusbreischluck

Sie werden im Nachtdienst zu einem 50-jährigen Pateinten gerufen. Vor 3 Tagen war eine Sigmaresektion aufgrund einer rezidivierenden Sigmadivertikulitis bei ihm erfolgt. Er stellt sich jetzt, nachdem es ihm die letzten Tage gut ging, zunehmend verwirrt dar, ist tachykard und hat Fieber. Was ist Ihre Verdachtsdiagnose?

Er hat aufgrund des Stress im Krankenhaus ein Durchgangssyndrom.

Er hat eine neu aufgetretene Herzrhythmusstörung entwickelt.

Er hat einen neuen Schub der Divertikulitis.

Er zeigt Zeichen einer beginnenden Sepsis, als Hinweis auf eine mögliche Anastomoseninsuffizienz nach der Sigmaresektion.

Er hat sich mit COVID-19 im Krankenhaus angesteckt.

In welchem Intervall soll der Wechsel einer offenporigen Schwammdrainage bei der endoskopischen Unterdrucktherapie in der Regel erfolgen?

Täglich

Je nach Befund der Anastomose, in der Regel alle 3–4 Tage

Alle 7 Tage

Alle 2 Wochen

Belassen bis zum vollständigen Verschluss der Leckage

Lässt sich die Anastomosenheilung nach Ösophagusresektion mit der präemptiven endoskopischen Unterdrucktherapie günstig beeinflussen?

Nein

Ja, eine primäre Abheilungsrate bis zu 20 % ist erreichbar

Ja, eine primäre Abheilungsrate bis zu 75 % ist erreichbar in Kombination mit einer enteralen Ernährung

Ja, eine primäre Abheilungsrate bis zu 100 % wurde in einer kleinen Patientenserie erreicht

Ja, aber nur in Kombination mit einer frühzeitigen Reoperation
